# Carnosine Content in Skeletal Muscle Is Dependent on Vitamin B6 Status in Rats

**DOI:** 10.3389/fnut.2015.00039

**Published:** 2016-01-19

**Authors:** Sofya Suidasari, Jan Stautemas, Shinji Uragami, Noriyuki Yanaka, Wim Derave, Norihisa Kato

**Affiliations:** ^1^Graduate School of Biosphere Science, Hiroshima University, Higashi-Hiroshima, Japan; ^2^Department of Movement and Sports Sciences, Ghent University, Ghent, Belgium

**Keywords:** vitamin B6, carnosine, skeletal muscles, rats, human volunteers

## Abstract

Carnosine, a histidine-containing dipeptide, is well known to be associated with skeletal muscle performance. However, there is limited information on the effect of dietary micronutrients on muscle carnosine level. Pyridoxal 5′-phosphate (PLP), the active form of vitamin B6, is involved in amino acid metabolisms in the body as a cofactor. We hypothesized that enzymes involved in β-alanine biosynthesis, the rate-limiting precursor of carnosine, may also be PLP dependent. Thus, we examined the effects of dietary vitamin B6 on the muscle carnosine content of rats. Male and female rats were fed a diet containing 1, 7, or 35 mg pyridoxine (PN) HCl/kg for 6 weeks. Carnosine in skeletal muscles was quantified by ultra-performance liquid chromatography coupled with tandem mass spectrometry. In the gastrocnemius muscle of male rats, carnosine concentration was significantly higher in the 7 and 35 mg groups (+70 and +61%, respectively) than in the 1 mg PN HCl/kg group, whereas that in the soleus muscle of male rats was significantly higher only in the 7 mg group (+43%) than in the 1 mg PN HCl/kg group (*P* < 0.05). In both muscles of female rats, carnosine concentration was significantly higher in the 7 and 35 mg groups (+32 to +226%) than in the 1 mg PN HCl/kg group (*P* < 0.05). We also found that, compared to the 1 mg group, β-alanine concentrations in the 7 and 35 mg groups were markedly elevated in gastrocnemius muscles of male (+153 and +148%, respectively, *P* < 0.05) and female (+381 and +437%, respectively, *P* < 0.05) rats. Noteworthy, the concentrations of ornithine in the 7 and 35 mg groups were decreased in gastrocnemius muscles of male rats (−46 and −54%, respectively, *P* < 0.05), which strongly inversely correlated with β-alanine concentration (*r* = −0.84, *P* < 0.01). In humans, 19% lower muscle carnosine content was found in soleus muscle of women of the lower plasma PLP tertile, but this was not observed in gastrocnemius muscle or in men. We conclude that adequate dietary vitamin B6 is essential for maintaining carnosine in skeletal muscles of rats. Significantly lower soleus carnosine content among women close to PLP deficiency suggests that a similar phenomenon exists in the humans.

## Introduction

Skeletal muscle contains at least 80% of the vitamin B6 pool in the body as pyridoxal 5′-phosphate (PLP) bound to glycogen phosphorylase. This information led to the studies on the effect of vitamin B6 status on exercise. A study by Richardson and Chenman suggested that vitamin B6 intake increased stamina as a result of prolonging muscle contraction (1). A case study demonstrated that vitamin B6 supplementation is beneficial for McArdle disease, a glycogenetic myopathy, especially for alleviating fatigue (2). A clinical trial also showed that a combined restricted intake of thiamine, riboflavin, and vitamins B6 and C causes a decrease in physical performance in humans (3). However, another study suggested that vitamin B6 supplementation, far higher than minimum daily requirement, had no positive effect on the exercise performance of rats (4). On the other hand, plasma PLP concentration is reported to increase after exercise (5). Thus, the role of vitamin B6 in skeletal muscle still remains unclear.

Pyridoxal 5′-phosphate, the active form of vitamin B6, acts as a cofactor for several enzymes involved in amino acid metabolisms. Some reports indicate that vitamin B6 deficiency modulates free amino acids levels in blood and tissues (6–8). Some enzymes modulating β-alanine metabolism, the precursor of carnosine, are PLP dependent. Recently, we reported that insufficient dietary vitamin B6 lowers the concentrations of carnosine and anserine in the heart of rats (9). Carnosine (beta-alanyl-l-histidine) and its methylated analog anserine are abundant dipeptides in skeletal muscle. They constitute an integral part of skeletal muscle contractility and homeostasis, presumably through their role as anti-oxidant, pH-buffering, anti-glycation and/or calcium regulator (10, 11). We hypothesized that dietary vitamin B6 plays an important role in maintaining muscle carnosine and anserine. Accordingly, this study investigated the effects of a low or high vitamin B6 diet on the levels of these metabolites and the related metabolites, such as β-alanine and ornithine, in the skeletal muscles of rats. Here, we report evidence that insufficient vitamin B6 intake lowers concentration of carnosine, which is a putative ergogenic factor, in the gastrocnemius and soleus muscles of male and female rats. In order to explore the relevance of our findings to humans, we additionally investigated the possible relationship between vitamin B6 status and muscle carnosine content in human volunteers.

## Materials and Methods

### Animal Study

#### Animals and Diets

Male and female Sprague–Dawley rats (3 weeks old, Charles River Japan, Hino, Japan) were maintained in accordance with the Guide for the Care and Use of Laboratory Animals established by Hiroshima University. Rats were housed in metal cages in a temperature controlled room (24 ± 1°C) and a 12-h light/dark cycle (lights on, 0800–2000 h). Rats had free access to food and deionized water. Rats were fed a commercial non-purified diet (MF, Oriental Yeast, Tokyo, Japan) for 1 week. In experiment 1, 24 male rats (average, 113 g) were divided randomly into three groups receiving 1, 7, or 35 mg Pyridoxine (PN) HCl/kg diet (*n* = 8/group) for 6 weeks. In experiment 2, 24 female rats were treated in the same way (average, 109 g). The basal diet comprised the components as described previously (12). PN HCl was supplemented to the basal diet at 1, 7, or 35 mg/kg diet. The level of PN HCl/kg diet recommended in the AIN-93 diet is 7 mg/kg (13). Meanwhile, 1 mg PN HCl/kg diet is reported to be the minimum level required for preventing growth depression caused by vitamin B6 deficiency (14). The animals were sacrificed by decapitation under diethyl ether anesthesia. Serum was collected and stored at −60°C. Gastrocnemius and soleus muscles were quickly dissected, frozen in liquid nitrogen, and immediately stored at −80°C until analysis.

#### Analysis of PLP in Serum and Skeletal Muscles by HPLC

The experimental methods were done as describe before (12). Briefly, vitamin B6 from serum was extracted using 3 N perchloric acid. PLP was converted to pyridoxic acid 5′-phosphate and measured by HPLC with a fluorometric detector (15). Muscles supernatant of each two rats from the same group were combined to obtain pooled samples for the analysis (*n* = 4/group). PLP in the pooled samples was measured in the same method (15).

#### Analysis of Carnosine, Anserine, and β-Alanine in Skeletal Muscles by UPLC-MS/MS Method

The experimental methods were done as describe before (9). Briefly, gastrocnemius and soleus muscles were treated with cold 3% sulfosalicylic acid to precipitate the proteins. The supernatant liquid of each two rats from the same group were combined to obtain pooled samples for the analysis (*n* = 4/group). The samples from male and female rats are represented in experiments 1 and 2, respectively.

#### Analysis of Ornithine in Skeletal Muscles by an Amino Acid Analyzer

The same pooled samples for the ultra-performance liquid chromatography coupled with tandem mass spectrometry (UPLC-MS/MS) analysis were used for ornithine analysis (*n* = 4/group). Ornithine concentration was quantified by an amino acid analyzer (JLC-500; JEOL, Tokyo, Japan). Amino acids mixture standard solutions (type AN-2 and type B) were used as standard solution (Wako, Osaka, Japan).

### Human Study

Human data were obtained from 24 male (age 20.6 ± 0.12 years) and 58 female (age 24.0 ± 0.12 years) recreationally active and healthy individuals. The study protocols were approved by the local ethical committee (Ghent University Hospital, Belgium) and written informed consent was obtained from all participants prior to the study.

Muscle carnosine concentration was determined non-invasively by proton magnetic resonance spectroscopy (1H-MRS) by means of a 3-T whole-body MRI scanner (Siemens Trio, Erlangen, Germany) as described by Baguet et al.(16). Muscle carnosine was determined in the soleus and gastrocnemius muscle of the right leg.

Venous blood samples were collected from an antecubital vein in EDTA tubes, immediately centrifuged after which the plasma was immediately stored at −80°C before analysis. Pre-analytical handling was performed in light-protected vials as described in the Chromsystem manual. PLP concentration was determined using a commercial HPLC-fluorescence method (52000/Premix, Chromsystems instruments & chemicals GmbH, Germany) (17). Non-fasted participants were in rest before and during the determination of muscle carnosine and at the time of blood withdrawal.

### Statistical Analysis

Data are expressed as means ± SE. Tukey’s multiple-range test was used to compare means after one-way ANOVA. Statistical significance of the difference among means was estimated at *P* < 0.05. Data analysis was performed using Excel Statistics 2010 for Windows (Social Survey Research Information Co., Ltd., Tokyo, Japan). Some data underwent regression analysis and the correlation coefficient was calculated.

The relationship between human plasma PLP levels and muscle carnosine levels was analyzed separately for men and women, given the known sex difference in muscle carnosine (16). Within each sex, subjects were divided into tertiles based on plasma PLP levels. Differences between tertiles were analyzed by ANOVA or Kruskal–Wallis depending on the results of the Levene test. *Post hoc* analyses were performed using Tukey or Wilcoxon tests. Relationships between some variables for both human and rodent studies were investigated by regression analysis and the Pearson coefficient determined (IBM Corp. Released 2013. IBM SPSS Statistics for Windows, Version 22.0. Armonk, NY: IBM Corp).

## Results

### Animal Study

#### Experiment 1 (Male Rats)

##### Food Intake, Body Weight, and Skeletal Muscles Weight of Male Rats

Dietary manipulation did not affect food intake, final body weight, and weight of skeletal muscles (*P* > 0.05 by ANOVA analysis, Table 1).

**Table 1:** **Body weight, food intake, muscles weight, and muscles PLP**.

PN HCl/kg	1 mg	7 mg	35 mg
**Experiment 1 (male rats)**			
Initial body weight (g)	113 ± 2	113 ± 1	113 ± 2
Final body weight (g)	434 ± 23	452 ± 12	461 ± 12
Food intake (g/6 week)	738 ± 40	793 ± 29	768 ± 18
Gastrocnemius muscle weight (g)	2.57 ± 0.08	2.47 ± 0.06	2.58 ± 0.14
Gastrocnemius muscle PLP (nmol/g)	9.1 ± 0.7^a^	12.8 ± 0.6^b^	12.8 ± 0.8^b^
Soleus muscle weight (g)	0.18 ± 0.01	0.17 ± 0.01	0.19 ± 0.01
Soleus PLP (nmol/g)	2.7 ± 0.3^a^	4.8 ± 0.2^b^	5.3 ± 0.1^b^
**Experiment 2 (female rats)**			
Initial body weight (g)	108 ± 2	109 ± 2	109 ± 2
Final body weight (g)	253 ± 5^a^	276 ± 9^a,b^	288 ± 8^b^
Food intake (g/6 week)	583 ± 13	630 ± 18	638 ± 16
Gastrocnemius muscle weight (g)	1.58 ± 0.04	1.73 ± 0.06	1.75 ± 0.07
Gastrocnemius muscle PLP (nmol/g)	7.6 ± 0.8^a^	14.5 ± 1.4^b^	14.5 ± 0.6^b^
Soleus muscle weight (g)	0.11 ± 0.00	0.12 ± 0.01	0.13 ± 0.01
Soleus muscle PLP (nmol/g)	2.0 ± 0.1^a^	5.1 ± 0.4^b^	5.4 ± 0.2^b^

*Mean ± SE (*n* = 7–8)*.

*^a,b^Means are significantly different by Tukey’s multiple-range test (*P* < 0.05)*.

##### PLP Concentrations in Serum and Skeletal Muscles of Male Rats

Serum PLP concentration increased in dose-dependent manner by vitamin B6 supplementation (0.17 ± 10, 0.55 ± 0.03, and 1.08 ± 0.07 μmol/L in the 1, 7, and 35 mg PN HCl/kg groups, *P* < 0.01 by ANOVA analysis). In gastrocnemius muscle, both the 7 and 35 mg groups showed +41% higher concentration of PLP (*P* < 0.05, Table 1) when compared to the 1 mg PN HCl/kg group. In soleus muscle, compared to the 1 mg PN HCl/kg group, the 7 and 35 mg groups had +81 and +100%, respectively higher concentration of PLP (*P* < 0.05, Table 1), without any differences between these groups (*P* > 0.05).

##### Concentrations of Carnosine, Anserine, *β*-Alanine, and Ornithine in Skeletal Muscles of Male Rats

**Figure 1:**
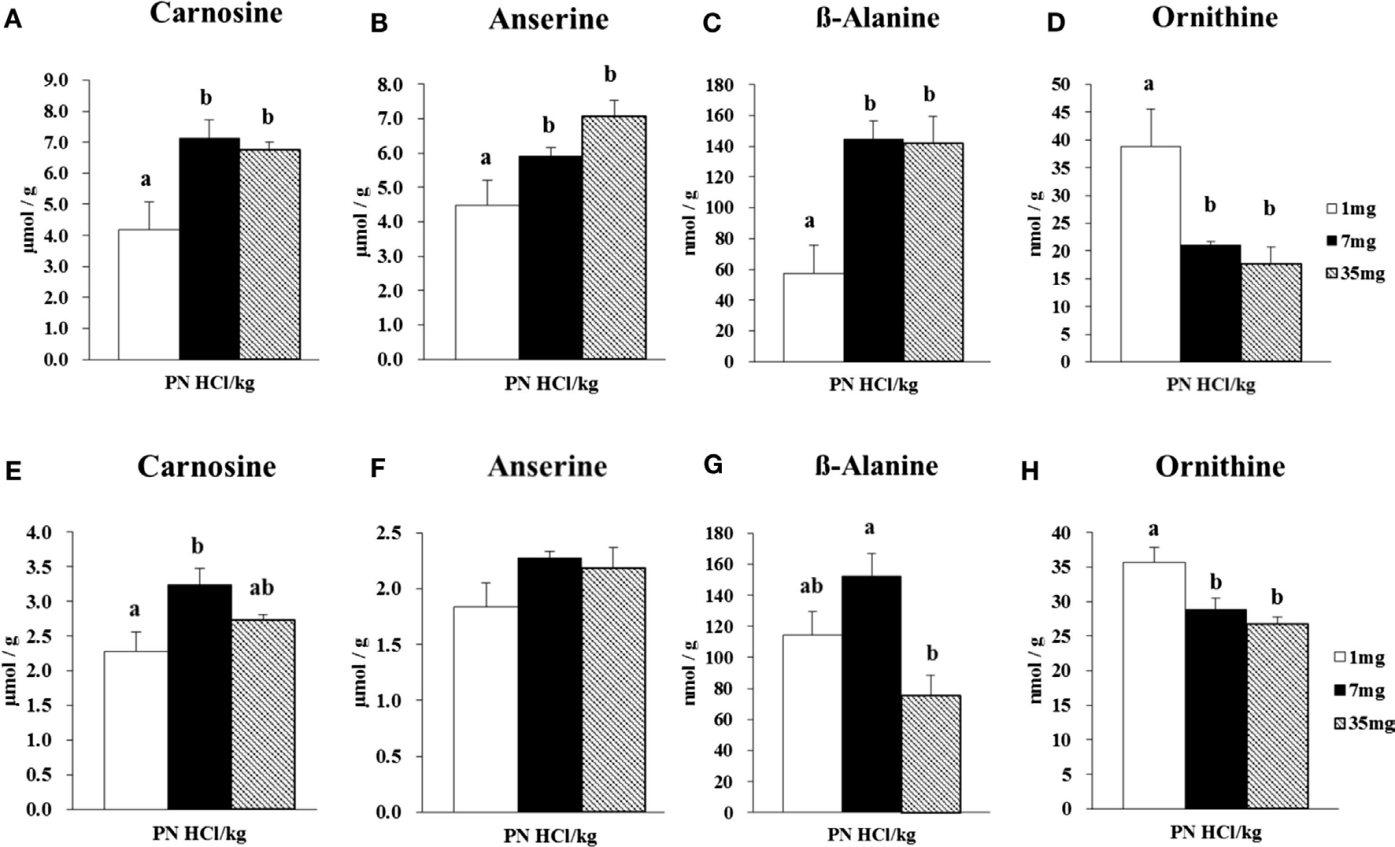
**Effect of dietary vitamin B6 levels on the concentrations of carnosine, anserine, β-alanine, and ornithine in gastrocnemius and soleus muscles of male rats**. Gastrocnemius muscle samples from male rats (*n* = 4) were analyzed for its concentrations of carnosine **(A)**, anserine **(B)**, β-alanine **(C)**, and ornithine **(D)**. Soleus muscle samples from male rats (*n* = 4) were analyzed for its concentrations of carnosine **(E)**, anserine **(F)**, β-alanine **(G)**, and ornithine **(H)**. Values are means ± SE. Groups with different letters are significantly different from each other, *P* < 0.05 (Tukey’s multiple-range test).

In the gastrocnemius muscle, the 7 and 35 mg groups had higher concentrations of carnosine (+70 and +61%, respectively, *P* < 0.05, Figure 1A) than that in the 1 mg PN HCl/kg group. The concentration of anserine was also higher (+58%) in 35 mg group when compared to 1 mg PN HCl/kg group (*P* < 0.05, Figure 1B). Carnosine and anserine concentrations were strongly correlated (*r* = 0.80, *P* < 0.01). The concentration of β-alanine was markedly higher in the 7 and 35 mg groups than that in the 1 mg PN HCl/kg group (+153 and +148%, respectively, *P* < 0.05, Figure 1C). There was a strong correlation between carnosine and β-alanine concentrations (*r* = 0.89, *P* < 0.01). Concentrations of carnosine, anserine, and β-alanine in the gastrocnemius muscle did not differ between the 7 and 35 mg PN HCl/kg groups.

Analysis using amino acid analyzer indicated that, in the gastrocnemius muscle, the concentration of ornithine was significantly lower in the 7 and 35 mg groups than the 1 mg PN HCl/kg group (−46 and −54%, respectively, *P* < 0.05, Figure 1D). Ornithine concentration was inversely correlated with β-alanine concentration (*r* = −0.84, *P* < 0.01).

In the soleus muscle, the 7 mg group had higher concentration of carnosine (+43%) than that in the 1 mg PN HCl/kg group (*P* < 0.05, Figure 1E), but the 35 mg group did not. There was no difference in the concentration of anserine among these groups (*P* > 0.05, Figure 1F). However, carnosine and anserine concentrations showed a significant correlation (*r* = 0.70, *P* < 0.05). The concentration of β-alanine did not differ between 1 and 35 mg groups (*P* > 0.05, Figure 1G), but lower in 35 mg group when compared to 7 mg group (−34%, *P* < 0.05, Figure 1G). There was no significant correlation between carnosine and β-alanine concentrations (*r* = 0.45, *P* > 0.05).

In the soleus muscle, the concentration of ornithine was significantly lower in the 7 and 35 mg groups than the 1 mg PN HCl/kg group (−19 and −25%, respectively, *P* < 0.05, Figure 1H). There was no significant correlation between ornithine and β-alanine concentrations (*r* = 0.32, *P* > 0.05).

#### Experiment 2 (Female Rats)

##### Food Intake, Body Weight, and Skeletal Muscles Weight of Female Rats

Dietary manipulation did not affect food intake and skeletal muscles weight (*P* > 0.05 by ANOVA analysis, Table 1). Compared to the 1 mg group, 35 mg PN HCl/kg group showed slightly higher final body weight in female rats (*P* < 0.05 by Tukey’s multiple-range test, Table 1).

##### PLP Concentrations in Serum and Skeletal Muscles of Female Rats

Serum PLP concentration increased in dose-dependent manner by vitamin B6 supplementation (0.05 ± 0.01, 0.45 ± 0.03, and 0.73 ± 0.07 μmol/L in the 1, 7, and 35 mg PN HCl/kg groups, *P* < 0.01 by ANOVA analysis). In gastrocnemius muscle, both 7 and 35 mg groups showed +91% higher concentration of PLP (*P* < 0.05, Table 1) when compared to 1 mg PN HCl/kg group. In soleus muscle, compared to the 1 mg group, the 7 and 35 mg PN HCl/kg groups had higher concentration of PLP (+156 and +172%, respectively, *P* < 0.05, Table 1).

##### Concentrations of Carnosine, Anserine, β-Alanine, and Ornithine in Skeletal Muscles of Female Rats

**Figure 2:**
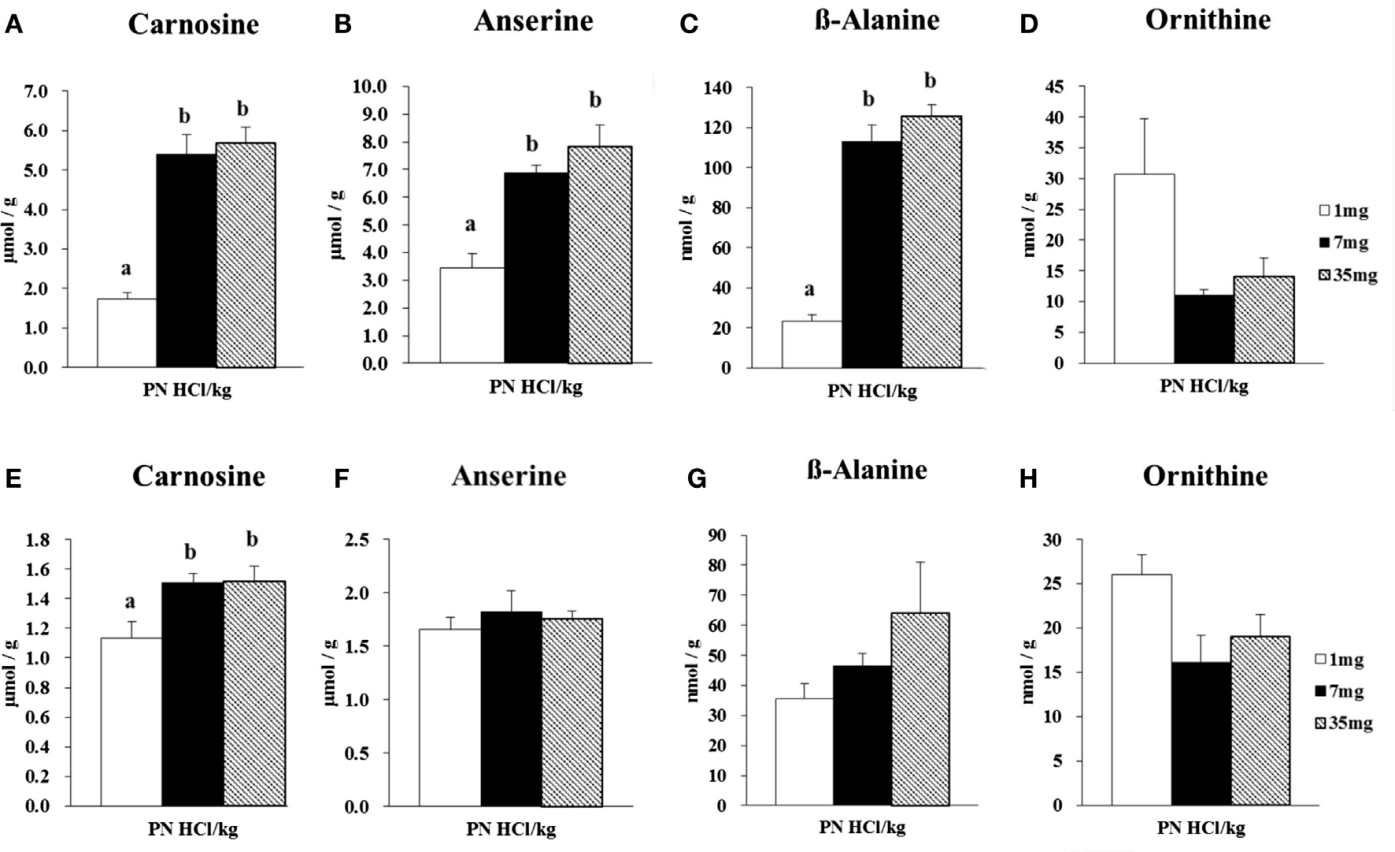
**Effect of dietary vitamin B6 levels on the concentrations of carnosine, anserine, β-alanine, and ornithine in gastrocnemius and soleus muscles of female rats**. Gastrocnemius muscle samples from female rats (*n* = 4) were analyzed for its concentrations of carnosine **(A)**, anserine **(B)**, β-alanine **(C)**, and ornithine **(D)**. Soleus muscle samples from female rats (*n* = 4) were analyzed for its concentrations of carnosine **(E)**, anserine **(F)**, β-alanine **(G)**, and ornithine **(H)**. Values are means ± SE. Groups with different letters are significantly different from each other, *P* < 0.05 (Tukey’s multiple-range test).

In the gastrocnemius muscle, the 7 and 35 mg groups had markedly higher concentrations of carnosine (+211 and +226%, respectively, *P* < 0.001, Figure 2A) and anserine (+100 and +128%, respectively, *P* < 0.01, Figure 2B), than those in the 1 mg PN HCl/kg group. Carnosine and anserine concentrations were strongly correlated (*r* = 0.89, *P* < 0.01). The concentration of β-alanine was remarkably higher in the 7 and 35 mg groups than that in the 1 mg PN HCl/kg group (+381 and +437%, respectively, *P* < 0.001, Figure 2C). There was a strong correlation between carnosine and β-alanine concentrations (*r* = 0.96, *P* < 0.01). Concentrations of carnosine, anserine, and β-alanine in the gastrocnemius muscle did not differ between the 7 and 35 mg PN HCl/kg groups.

In the gastrocnemius muscle, the concentration of ornithine tended to be lower in the 7 and 35 mg groups, −64 and −54% respectively, than the 1 mg PN HCl/kg group (*P* = 0.066 by ANOVA analysis, Figure 2D). Ornithine concentration was inversely correlated with β-alanine concentration (*r* = −0.65, *P* < 0.05).

In the soleus muscle, the 7 and 35 mg groups had significantly higher concentrations of carnosine (+156 and +172%, respectively, *P* < 0.001, Figure 2E), when compared to the 1 mg PN HCl/kg group. There were no significant differences in the concentrations of anserine and β-alanine among the three groups (*P* > 0.05, Figures 2F,G). There was no significant correlation between carnosine and β-alanine concentrations (*r* = 0.56, *P* > 0.05).

In the soleus muscle, the concentration of ornithine tended to be lower in the 7 and 35 mg groups, −64 and −54% respectively, than the 1 mg PN HCl/kg group (*P* = 0.069 by ANOVA analysis, Figure 2H). There was no significant correlation between ornithine and β-alanine concentrations (*r* = −0.01, *P* > 0.05).

#### Human Study (Experiment 3)

##### Concentrations of Plasma PLP and Muscle Carnosine in Humans

In the human cohort, plasma PLP concentrations ranged between 25.2 and 203.9 nmol/L in women, and between 52.2 and 571.3 nmol/L in men. No individuals could be termed deficient (<20 nmol/L) and only one had insufficient (<30 nmol/L) plasma PLP (25.2 nmol/L). In the latter subject, the carnosine concentrations in soleus and gastrocnemius muscle were, respectively, 2.72 and 3.21 μmol/g tissue, which is −0.19 and −0.63 *z*-score below the average of our reference database containing 90 women.

**Figure 3:**
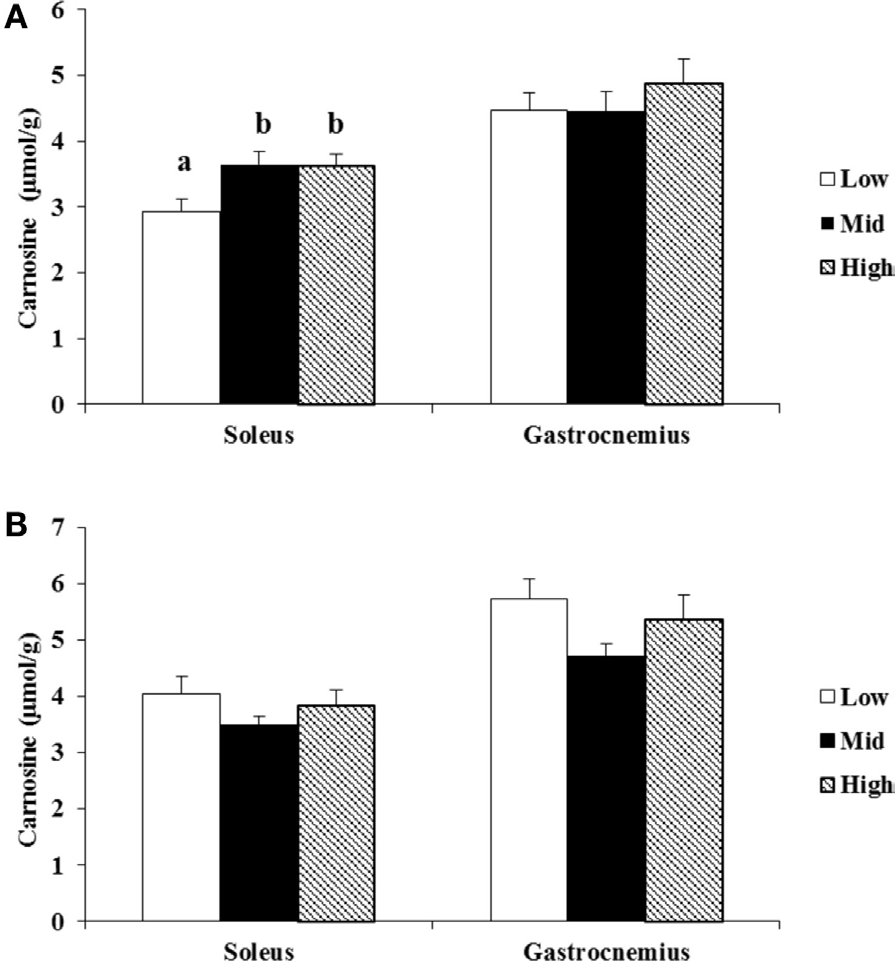
**Association of plasma PLP with carnosine concentrations in different tertiles of women (A) and men (B)**. Values are means ± SE. Groups with different letters are significantly different from each other, *P* < 0.05 (Tukey’s multiple-range test).

The known difference in carnosine concentrations between men and women (18) was confirmed in this cohort (*P* < 0.05). Therefore, tertiles for PLP were calculated separately for both sexes. Carnosine concentrations were 19.5 (*P* < 0.05) and 18.8% (*P* < 0.05) lower in the lowest tertile of the soleus of women compared to mid and high tertile (Figure 3A). No other differences (women gastrocnemius, or men soleus and gastrocnemius) were found (Figures 3A,B). In women, a positive correlation of *r* = 0.31 and *r* = 0.29 was established between plasma PLP and, respectively, soleus and gastrocnemius muscles (*P* < 0.05 and *P* < 0.05). In men, a correlation in the soleus (*r* = 0.41, *P* < 0.05), but not in the gastrocnemius (*r* = 0.04, *P* > 0.05), muscle was found. The male cohort contained one outlier (plasma PLP = 570 nmol/L). After deletion of this outlier, no correlation was found in either muscle (respectively, for soleus and gastrocnemius *r* = −0.23 and −0.10; *P* > 0.05).

## Discussion

This study demonstrated that dietary vitamin B6 supplementation (i.e., 7 and/or 35 mg PN HCl/kg) compared to a low-vitamin B6 diet (i.e., 1 mg PN HCl/kg) significantly increased the concentrations of carnosine in the skeletal muscles of male and female rats. Meanwhile, dietary supplemental vitamin B6 appeared to affect the anserine concentrations to a lesser extent. An elevated muscle carnosine and anserine content exerts beneficial effects on the skeletal muscle contractility, as shown in isolated human muscle fibers (19), isolated rodent muscles (20), and *in vivo* human muscles (18). The underlying ergogenic mechanism probably relates to the dipeptides’ capacity as an anti-oxidant, pH-buffer, and/or calcium regulator (10, 21). Thus, the present results imply that maintaining recommended amounts of dietary vitamin B6 might be favorable for skeletal muscles function by elevating the histidyl-dipeptides, especially carnosine.

The present study shows that the dependency of carnosine concentration on dietary vitamin B6 intake was more remarkable in the gastrocnemius than the soleus muscle in both male and female rats. Oppositely, in humans, the link between vitamin B6 status and muscle carnosine was only found in (female) soleus, not gastrocnemius. The gastrocnemius muscle comprises a high proportion of type II myofibers, which are “fast-twitch” fibers; meanwhile, the soleus muscle comprises predominantly type I myofibers, which are characteristically “slow-twitch” fibers. On the other hand, compared to the male rats, the female rats appeared to show more remarkable increase in carnosine concentration by dietary B6 supplementation in gastrocnemius muscle. Thus, the effects of vitamin B6 status on carnosine concentrations in skeletal muscles appear to differ with respect to muscle fiber type, gender, species, and the interaction of these factors. However, the reason for this is unknown.

Dietary supplementation of carnosine and β-alanine is reported to increase carnosine concentration in the skeletal muscles (22, 23), whereas a histidine-deficient diet can reduce carnosine concentration (24). However, those studies involve chronic dietary intervention of high doses of carnosine and β-alanine, or severe histidine-deficiency conditions (22–24). By contrast, the present study used diets ranging from a low-vitamin B6 diet (marginal vitamin B6-deficient diet) that did not induce severe vitamin B6 deficiency to somewhat higher vitamin B6 diets far below the supraphysiological dose (i.e., acute toxic dose). To our knowledge, this is the first evidence indicating dietary intake of a nutrient close to the range of regular daily intake plays a pivotal role in maintaining carnosine concentration in skeletal muscles.

In order to find out whether the rodent data could be replicated in humans, we measured carnosine and PLP in volunteers. We divided the female and male participants separately into tertiles in order to try to mimic the three conditions in the rats. However, the lowest tertile was not as insufficient as the rats in the 1 mg PN HCl/kg group were. Still, we found that women have significantly reduced muscle carnosine content in the lowest PLP tertile compared to the higher tertiles. Moreover this was somewhat confirmed by the low, but significant correlation. In men, these results could not be replicated. This is probably due to the fact that the lowest tertile in men was not as close to the insufficient range as the women were. We can conclude from the human data in this study that to some extent a similar pattern could be recognized as in the rats, albeit only in women. Yet, it is expected that the vitamin B6 dependency would probably be more pronounced if the study group would have included truly vitamin B6-deficient humans. Obviously, further study is necessary to explore the direct relationship between muscle carnosine concentration and vitamin B6 status in an interventional design, since this cross-sectional study cannot exclude confounding variables.

In the rat study, it is noteworthy that the concentration of β-alanine, a precursor of carnosine, in gastrocnemius muscles was markedly elevated by supplemental vitamin B6. Likewise, we consistently found a strong correlation between β-alanine and carnosine concentrations in this muscle. It remains to be determined which PLP-dependent enzymes are responsible for the changes in carnosine concentrations. β-Alanine is the rate-limiting precursor of carnosine (25). The most important source of β-alanine is uracil degradation, but the enzymes involved are not PLP dependent. Enzymes that might be responsible are PLP-dependent transaminases “β-alanine – 2-oxoglutarate transaminase” (ABAT) and/or “β-alanine – pyruvate transaminase” (AGXT2). Animal study suggested β-alanine as one of the metabolites resulted from the injection of spermidine or spermine, which are produced by PLP-dependent ornithine decarboxylase from ornithine (26, 27). Interestingly, our study indicated significant inverse correlation between ornithine and β-alanine concentrations in gastrocnemius muscle, which shows more remarkable response of β-alanine to dietary vitamin B6 compared to soleus muscle. This raises the possibility that the elevation in the level of β-alanine in the gastrocnemius muscle by dietary supplemental vitamin B6 is at least in part mediated by enhancing the conversion from ornithine to β-alanine in skeletal muscles. Further study is necessary to test this possibility.

In conclusion, the results of this study suggest that dietary vitamin B6 status is a determinant of carnosine concentration in laboratory rat skeletal muscles. The present study also suggests that vitamin B6 intake close to the range of regular daily intake is able to affect carnosine concentration in skeletal muscles. The link observed between plasma PLP and muscle carnosine in human subjects suggest a similar phenomenon likely exist in human. As histidine-containing dipeptides are critical for muscle function, this is a novel mechanism why adequate vitamin B6 status is essential to safeguard optimal muscle contractility and exercise performance.

## Author Contributions

SS and NK designed the research; SS, JS, SU, and NY conducted the research; SS and JS analyzed the data; and SS, JS, WD, and NK wrote the paper and had primary responsibility for the final content. All authors read and approved the final manuscript.

## Conflict of Interest Statement

The authors declare that the research was conducted in the absence of any commercial or financial relationships that could be construed as a potential conflict of interest.

## Abbreviations

PLP, pyridoxal 5′-phosphate; PN, pyridoxine; UPLC-MS/MS, ultra-performance liquid chromatography coupled with tandem mass spectrometry.
